# Human serum albumin as a clinically accepted cell carrier solution for skin regenerative application

**DOI:** 10.1038/s41598-020-71553-2

**Published:** 2020-09-02

**Authors:** Hady Shahin, Moustafa Elmasry, Ingrid Steinvall, Katrin Markland, Pontus Blomberg, Folke Sjöberg, Ahmed T. El-Serafi

**Affiliations:** 1 The Department of Biomedical and Clinical Sciences (BKV), Linköping University Hospital, 401A, Building 462, Floor 11, P.O. Box 581 85, Linköping, Sweden; 2 Department of Hand Surgery and Plastic Surgery and Burns, Linköping University Hospital, Linköping, Sweden; 3 Faculty of Biotechnology, Modern Sciences and Arts University, Cairo, Egypt; 4 Vecura, Karolinska Cell Therapy Center, Karolinska University Hospital, Stockholm, Sweden; 5 Department of Laboratory Medicine, Clinical Research Center, Karolinska Institutet, Stockholm, Sweden; 6 Medical Biochemistry Department, Faculty of Medicine, Suez Canal University, Ismailia, Egypt

**Keywords:** Clinical trial design, Translational research

## Abstract

The rules governing Medicinal Products in the European Union necessitates the production of cell-based therapy in good manufacturing practice facilities. The produced cells may need several hours in transportation to reach the application sites. In this study, we investigated four candidate solutions for transporting human keratinocytes. The solutions are (1) normal saline, (2) saline with 2.5% human serum albumin (Saline + HSA), (3) chemically defined, xeno-free keratinocyte media and (4) keratinocyte media with pituitary bovine extract (PBE-media). One million keratinocytes from three donors were suspended in each solution and kept at 4 °C for up to 24 h. Cells kept in Saline + HSA showed higher viability after 1, 3 and 24 h. Then, equal number of viable cells were seeded on collagenous matrix and cultured for 48 h. The adhesion and colonization were higher in the cells kept in PBE-media, while the keratinocyte surface marker, cytokeratin 14, was present in all studied groups. These results confirmed the suitability of Saline + HSA as a cell transportation solution for clinical use, which will be the choice for the planned clinical trial. Keratinocyte PBE-media can be an alternative for cells transported for research purpose, if the same media type is going to be used in the following experiments.

## Introduction

The clinical applications of cellular therapy is emerging in many fields, including burn and chronic wound management^1^. Cultured autologous keratinocytes can provide an opportunity for faster healing and for better skin quality^2,3^. The field of cell therapy is generally considered as Advanced Therapy Medicinal Products, (ATMPs), which has several updated regulations in the last decade^4^. Thus, quality assurance and control of the production process are more stringent than standard cell culture. The cells have to be produced according to the ‘Guidelines on Good Manufacturing Practice (GMP) specific to Advanced Therapy Medicinal Products’, adopted by the European Commission in Nov 2017. GMP is usually available as a central facility, and many challenges are hence present. One example of ATMPs is keratinocytes that are sprayed on surgically excised burn wounds, in situations where patients suffer from major burns and lack enough intact skin donor sites that can be used for harvesting autologous skin grafts. In the national burn center in Linköping University Hospital in Sweden, treatment of major burns with autologous sprayed keratinocytes has been in use for more than 15 years. In the last decade, the European Union has set strict regulations to deliver cell based medicinal products. The production of ATMPs, including cultivation of cells, must be undertaken in fully GMP compliant environment. Consequently, the application of this treatment in Linköping has stopped, and the manufacture of the keratinocytes had to be updated to be compliant with these new regulations.

Following the production, the cells will need to be transported to the clinical site that will administer the cells. The transportation process can take several hours, based on the distance between the manufacturing and application sites. Furthermore, the readiness of the patient and the surgical team for application might require extra time, which can be extended up to 24 h. It is preferred to transport the cell product as fresh cells since freezing the cells can be associated with decreased cell numbers and phenotype. Furthermore, quality control could be required after thawing, which could affect the certified release criteria and cannot be conducted outside the manufacturer site^5–7^. The debate about the optimum cell carrier/transport solution was focused on maintaining the viability of the cells without affecting their phenotype or functionality, for this time span. This study evaluated four candidate solutions for transporting keratinocyte at 4 °C for 24 h, which included (1) normal saline solution (0.9% NaCl); (2) saline with 2.5% human serum albumin (Saline + HSA); (3) chemically defined, xenofree keratinocyte media (CDM), which will be used for keratinocyte expansion for clinical use; and (4) keratinocyte media with bovine pituitary extract (BPE-media), the classically used media for keratinocyte expansion in research.

## Results

### Keratinocytes viability following preservation at + 4 °C

**Figure 1:**
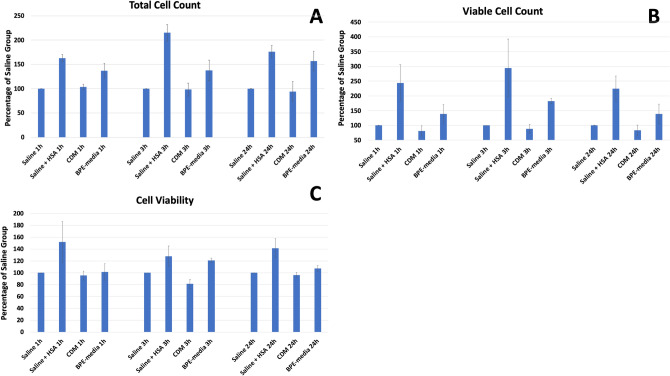
The cell counting and viability was related to that of the saline group in each sample. The total cell number (**A**) was higher in the saline + HSA group after 1 and 24 h in comparison to saline and CDM groups and higher than all studied groups at 3 h. BPE-media was higher in comparison to saline after 1 and 24 h and more than CDM at 24 h. The viable cell number (**B**) was similarly higher in saline + HSA in comparison to saline and CDM at all studied timepoints, with a similar trend with BPE-media. The cell viability (**C**) was higher in saline + HSA group in comparison to CDM at all timepoints and to saline at 24 h. BPE-media had higher viability than saline and CDM groups at 3 h. n = 3.

Keratinocytes were kept in the corresponding transport solution at + 4 °C for 24 h. The cell count and viability were determined after 1 h, 3 h and 24 h (Fig. 1). After 1 hour, cells suspended in saline + HSA showed higher total cell number (163% of the saline group) than the rest of the groups, while the number of viable cells was higher (244%) than among those suspended in saline (100%) and CDM (81%). Similarly, cells kept in Saline + HSA showed higher viability (152%) than CDM (95%) with a similar tendency with BPE-media (101%).

After 3 h, the number of viable cells was higher in Saline + HSA (295%) and BPE-media (182%) groups than the saline (100%) and CDM (89%) groups. The cell viability was lower in CDM than the rest of the groups. BPE-media had higher viability than the saline group and the cells suspended in Saline + HSA showed a similar tendency.

After 24 h, the number of viable cells (225%) as well as the cell viability (142%) were higher in the Saline + HSA group in comparison to the saline (100%; 100%) and CDM (83%; 96%) groups and with a similar tendency in comparison with BPE-media (139%; 107%).

Total cell number was higher in the Saline + HSA group in comparison to the saline and CDM groups at all studied timepoints, as well as BPE-media group after 3 h with a similar tendency after 1 h. The latter group had significant higher total cell number than saline after 1 h and 24 h and a similar tendency at 3 h. No difference was detected between the total cell number of the saline and CDM groups at any of the studied timepoints.

### Keratinocyte adhesion and colonization after preservation

**Figure 2:**
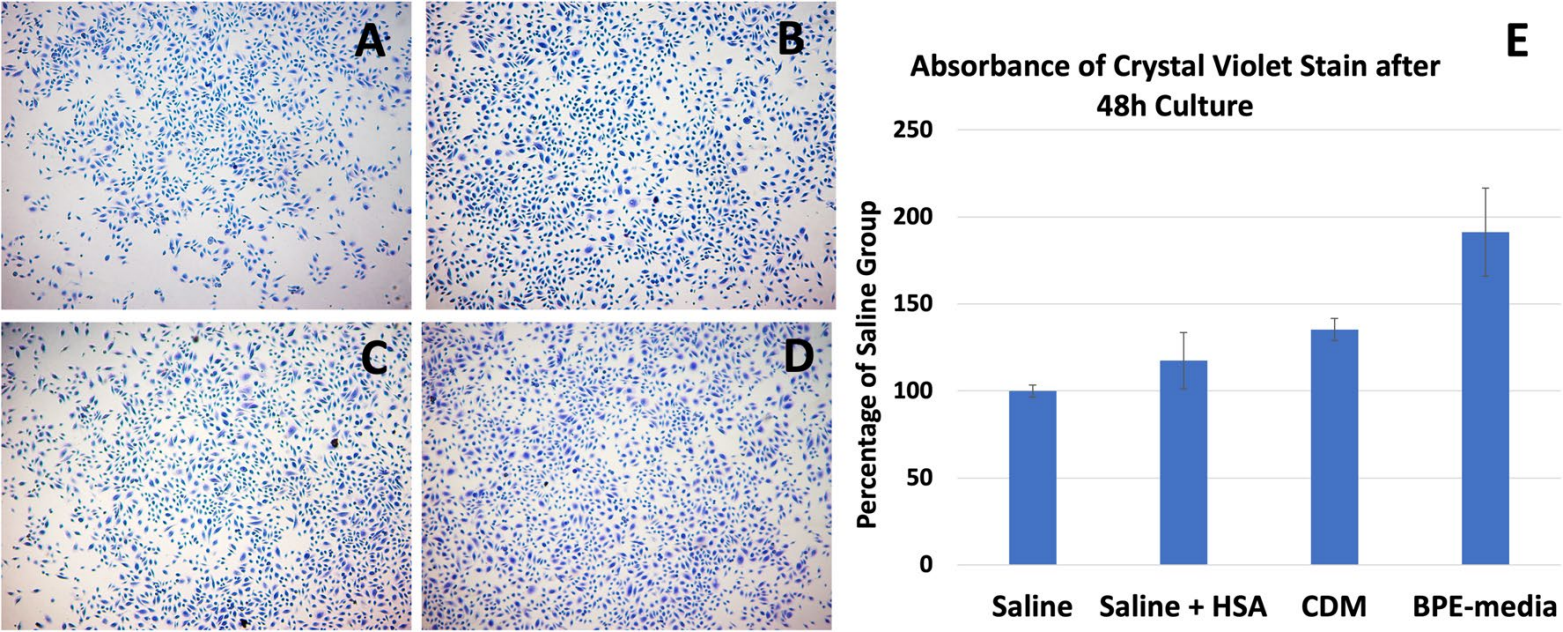
Crystal violet staining showed adherence and colonization, following 24 h incubation at 4 °C, of keratinocytes kept in saline (**A**), saline + HSA (**B**), CDM (**C**) and BPE-media (**D**). The elution of the stain (**E**) was higher in the BPE-media in comparison to the rest of the groups. Also, CDM had higher intensity than saline group.

After 24 h in the corresponding transport solution, the cells were resuspended and equal number of viable cells were cultured at 37 °C and 5% CO_2_, for 48 h. The cells were then fixed and stained with crystal violet (Fig. 2). The viable cells from all transport solutions kept their ability to adhere to the culture surface, as well as colony formation. While visual difference of the staining between the groups was not clear, elution of the stain showed higher absorbance for the cells in PBE-media (191%) in comparison to all other groups, as well as between CDM (135%) and saline (100%). The was no difference between CDM and saline + HSA (117%).

### Keratinocytes expression of the surface marker cytokeratin 14 and involucrin

**Figure 3:**
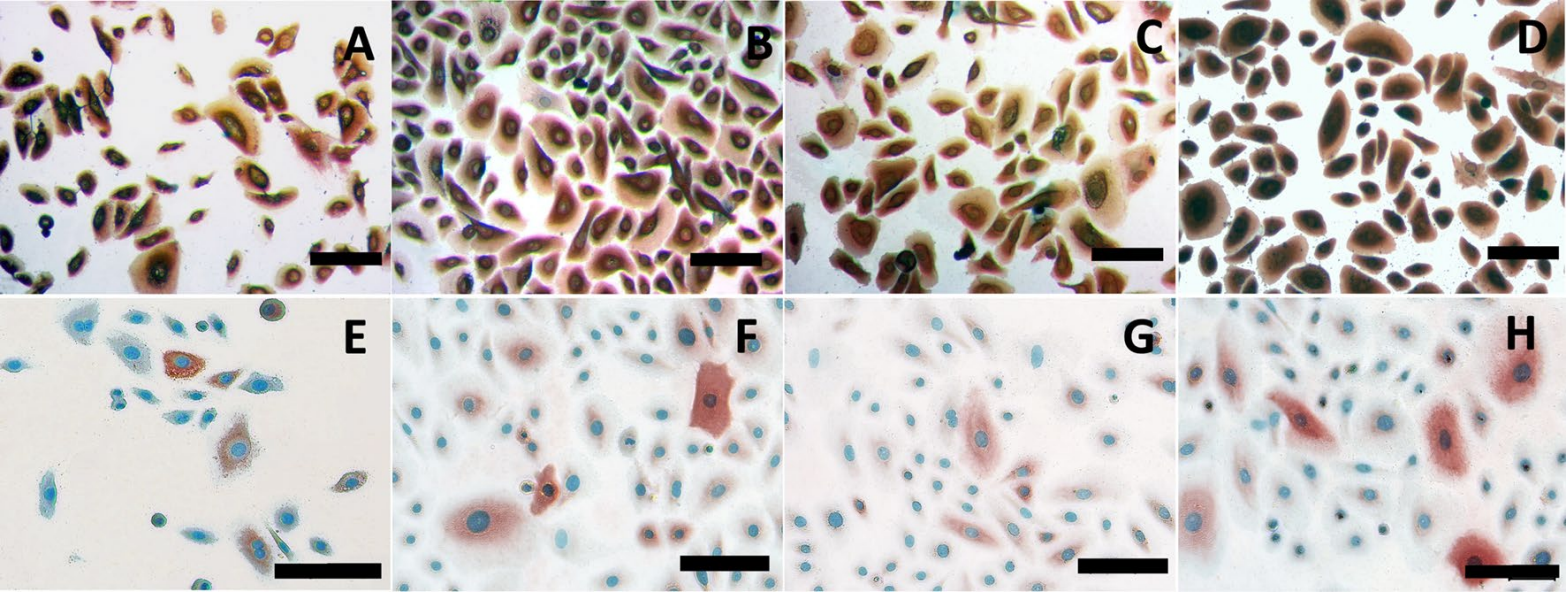
Immunocytochemical staining for cytokeratin 14 (**A**–**D**) and involucrin (**E**–**H**). Keratinocytes kept in saline (**A**, **E**), saline + HSA (**B**, **F**), CDM (**C**, **G**) and BPE-media (**D**, **H**) showed comparable positive staining for the studied markers. Scale bar was set at 100 µm.

Keratinocytes were fixed after 48 h culture and characterized by immuno-staining of the keratinocyte markers cytokeratin 14 (CK-14) or involucrin. The cells from all suspension groups were positive for the stain, and no difference could be observed between the groups (Fig. 3).

## Discussion

Cell based therapy is an upcoming field in modern medical management of many conditions, including skin wounds^8,9^. The European Directorate for the Quality of Medicines & Health Care has considered cultured cells as an advanced medicinal product, which needs extensive validation. The whole manufacturing process needs to comply to the regulations of the European Medicines Agency (EMA), including the transportation procedure. While the temperature and sterility chain are well described, the transport solution has yet to be established. This solution should provide a vehicle that maintain the cell phenotype, function and viability over the transportation time from the manufacturing to the application sites. Furthermore, the cells may sometimes have to be kept overnight when the application is not immediately possible. The transportation of cells is not only limited to ATMP or research but also for diagnostic purposes. In an interesting study, the authors shipped whole blood samples overnight, in their collection tubes, to a distant laboratory and investigated the possible effect on the immune cells. While T lymphocytes showed mild shift of their subclasses, B lymphocytes and natural killer cells had altered production of certain cytokines^10^.

In Linköping University Hospital, a plan is undergoing to treat burn patients and chronic wounds patients with autologous sprayed keratinocytes. The cells will be manufactured by the Vecura GMP facility in Stockholm and will need to be transferred to the premises in Linköping, about 200 km south-east of Stockholm. Ideally, the cells will be applied immediately but, according to the patient condition and the availability of the surgical team, a delay for up to 24 h could be expected. A multidisciplinary team had discussions regarding the best vehicle for transporting the cells and preserving them overnight. The suggestions included having a balanced solution, such as normal saline, or cell culture media. The later should have the advantage of providing necessary nutrients to the cells. Some laboratories transfer or exchange cells just by shipping living cells in fresh cell culture media, in sterile container and in ambient temperature. In this study site, two types of media are used for culturing keratinocytes; the first is described as serum free media which has bovine pituitary extract among other components. This media is not recommended to be used for manufacturing cells for clinical application due to the risk of transferring diseases between animals and humans. Instead, a xeno-free media is being used for such purpose. Saline is the most used balanced solution, that can be readily accessible as a authorized medicinal product. Lymphoid tissue was freshly transported in saline at refrigerator temperature and the immunophenotyping has been stable for 7 days^11^. In order to provide the saline with a protective vehicle, 2.5% of HSA was added to the solution. HSA was used as a substitute for fetal calf serum in cell microencapsulation in poly lysine alginate for biomedical application^12^. This composition is common for drug delivery solutions for injection^13^. In addition it has been used as a preservative solution, at the same concentration, for endothelial and smooth muscle cells^14^. In the latter study the authors investigated different concentrations of human serum albumin, in comparison to normal saline, to preserve the cells for 24 and 48 h at 4 °C. The authors concluded that 2.5% is the optimum concentration, as proved by the viability of the cells and their adherence after preservation. In another study, Lee et al. found that adding 10% HSA to saline or Dulbecco's Modified Eagle Medium would enhance the viability of gingival-derived oral keratinocytes when kept at 4 °C. The cells kept the same distribution among different phases of the cell cycle up to 48 h, but the authors did not show any functional or phenotypic effects. Interestingly, there was no difference in the cell viability when the cells were kept in saline with 10% HSA either in 4 °C or 25 °C^15^.

The production of recombinant HSA overcame the availability and potential infectivity problems that can be associated with this important protein. HSA is widely used as a balanced drug carrier in solutions, as well as in more complicated drug delivery systems^16,17^. Albumin is incorporated into the cell culture in conjunction with growth factors and other peptides. Additionally, this small protein can bind to the external surface of the cell membrane, through non-specific adsorption, and provides cell protection. This role is particularly evident in bioreactors, where albumin prevent the physical damage of the cells caused by hydrodynamic stress. Furthermore, albumin can be internalized to the cells by endocytosis, through clathrin-coated pits^12^. To the best of our knowledge, this effect has not been studied in keratinocytes.

Cell culture media was used as a transport solution for avulsed teeth, which can keep the viability of the tooth for 48 h. After reimplantation, the periodontal ligament cells were viable during 12 months of follow up^18^. Similarly, fibroblast sheets were transported in cell culture media for 8 h without thermal regulation (20–30 °C), on ice (5–10 °C) and in a special container that keeps the temperature at 37 °C, without significant difference in the histological morphology of the layers, in comparison to the control. The latter was kept in the standard CO_2_ incubator^19^. In a similar experiment, mucosal epithelial sheets were transported for 12 h in four different transportation media; keratinocyte culture media, keratinocyte culture media without fetal calf serum, DMEM/F12 media with epidermal growth factor and Hanks balanced salt solution. The viability of the cells upon delivery was 89.9%, 93.8%, 87.1% and 90.7%, which indicated no significant difference between the studied solutions. The morphology of the cell sheets was similar before and after transportation^20^. These reports confirmed the validity of the cell culture media in the transportation of living chilled cells, which matched our findings. Cells kept at 4 °C in cell culture media were still viable and able to adhere to cell culture plastic.

On the other hand, BPE was added as an essential component of keratinocytes culture media as BPE enhanced proliferation and helped to maintain their phenotype^21^. The protein component of BPE is cell-protective against oxidative stress. Its mitogenic ability was estimated to be 70 times higher than serum because of the presence of a cocktail of growth factor, such as growth hormone, basic fibroblast growth factor, epidermal growth factor like proteins, and platelet-derived growth factor^22^. In our results, the higher colonization ability with PBE-media group could reflect the effect of these factors. In this case, the safety of the PBE-media as a carrier solution would be questioned. Despite the excessive wash, the cells had higher ability to adhere and proliferate, which could reflect either binding of the bovine growth factors to the human cells or—at least—stimulating the corresponding signaling pathway(s). In the first case, there will be a risk of transmitting an animal protein to the patient. Hypersensitivity can be a consequence with failure of cell transplantation. Such effect is well known for patients received cultured cells in the presence of fetal calf serum^23,24^. Besides, the transmission of infectious agents from the animal derived raw materials to humans is a health risk, especially with prions^12^. In the second case of complete removal of animal-derived protein, the uncontrolled mitogenic stimulation of these pathways would need detailed investigations for their safety, on the short and long terms.

In conclusion, this study recommends the use of 2.5% HSA in saline as a transportation vehicle for keratinocytes for up to 24 h at 4 °C for clinical use. The research team decided, after several discussions, to use this solution for the cell transportation of the proposed clinical trial based on the perseverance of cell viability as well as the phenotype. Furthermore, the components of this solution can be obtained as authorized medicinal products that is compliant to EMA guidelines. The ultimate evaluation of our findings will be reflected by the success rate of keratinocyte application in clinical studies. In the meantime, keratinocyte media with PBE can be used for the transportation purpose, only if the cells will be used for research purpose and this type of media will be used in the downstream experiments.

## Methods

### Primary human keratinocytes isolation and culture

Keratinocytes were isolated from split thickness skin biopsies obtained from three healthy donors who underwent abdominoplasty and/ or mammoplasty surgeries at the Department of Hand Surgery and Plastic Surgery, Linköping University Hospital. The biopsies were anonymized. The study was approved by the Swedish Ethical Review Authority (no. 2015/177-31). All methods were performed in accordance with the relevant guidelines and regulations. Informed consent was obtained from all patients. Skin biopsies were processed at the Research and Development Unit for Skin and Cultured Cells, Linköping University Hospital. Briefly, skin was cut into 1–2 mm^2^ pieces and incubated with 10 mg/ml dispase II (Gibco, USA) in serum-free DMEM (Gibco, USA), overnight at 4 °C. The epidermis was peeled away and incubated for 30 min with 0.05% Trypsin–EDTA (Sigma, USA) on a rocking stage at 37 °C. Then the digestion was stopped by adding equal amount of DMEM media + 10% FBS (Gibco, USA). The solution was passed through 70 µm cell strainer with excess serum-free DMEM. Keratinocytes were expanded in keratinocyte serum free medium supplemented with epidermal growth factor, bovine pituitary extract BPE, and Gentamicin/Amphotericin Solution (Gibco, USA). Media was changed every second day.

### Preparation of ‘cell transportation’ solutions

Keratinocytes, passage 1–3, were trypsinized and suspended as 1 × 10^6^ of viable cells per ml, in 4 different solutions; (a) 0.9% NaCl for clinical use (Braun, Germany) as control, (b) 2.5% human serum albumin (Sigma-Aldrich, USA) in saline (Saline + HSA), (c) xenofree, chemically defined media (CDM) for keratinocyte (EpiLife, Thermo Fisher Scientific, USA) and (d) keratinocyte serum free medium (Keratinocyte SFM, Gibco, USA) supplemented with epidermal growth factor and bovine pituitary extract (BPE-media), supplied as a multicomponent kit. For each sample, three independent aliquots were prepared.

### Cell counting and viability

All the samples were kept at 4 °C and 1 ml aliquots of each sample were counted in triplicates at 1 hour (h), 3 h and 24 h. The cells were mixed as a 1:1 with 0.4% trypan blue and counted using the TC20 automated cell counter (Bio-Rad Inc, Hercules, California). The total number of cells, viable cells and viability percentage were recorded.

### Cell seeding

Keratinocytes kept for 24 h at 4 °C were seeded in 24-well plates, at a density of 2.5 × 10^4^ cell/cm^2^ in CDM, after coating the plate with the collagenous coating matrix according to the manufacturer’s instructions (Gibco, USA) and kept at 37 °C and 5% CO_2_ for 48 h. Then, the cells were fixed by ice-cold methanol for 15 min at − 20 °C and were left to air dry.

### Crystal violet assay

Fixed cells were rinsed with phosphate buffered saline (PBS) and incubated for 10 min at room temperature with 0.5% crystal violet solution (Sigma-Aldrich, USA). The cells were excessively washed with running water. Cell adherence and colonization was examined using an inverted microscope and imaged with an AxioCam ERc5s camera (Carl Zeiss Microscopy, Germany). Then the stain was eluted in 10% acetic acid (Sigma-Aldrich, USA) and intensity was evaluated spectrophotmetricaly at 595 nm (Spectramax plus 384, Molecular Devices, USA).

### Immunocytochemistry

The fixed keratinocytes underwent antigen retrieval using preheated tris-urea buffer (Thermo Scientific, USA) at 95 °C. The cells were permeabilized by incubation for 15 min at room temperature with 0.05% Triton X-100 (Thermo Scientific, USA). Following blockage, anti-cytokeratin 14 or anti-involucrin (Abcam, UK) at 1 μg/ml was incubated overnight on a rocking stage at 4 °C. Then the cells were washed, and the mouse and rabbit specific secondary antibody with horseradish peroxidase/3-amino-9-ethylcarbazole detection immunohistochemistry kit (Abcam, UK) was used according to the manufacturer’s instruction and counterstained with Alcian blue solution (Sigma-Aldrich, USA). The stained cells were imaged using inverted microscopy, as described above.

## Data Availability

The authors confirms the availability of data according to the Nature Research policies for the sharing of research materials.
